# Determining the effect of femoral anteversion and hip alignment parameters on lower limb and knee alignment post-total hip arthroplasty

**DOI:** 10.1038/s41598-025-30987-2

**Published:** 2025-12-02

**Authors:** Shunichi Yokota, Tomohiro Shimizu, Hotaka Ishizu, Daisuke Takahashi, Norimasa Iwasaki

**Affiliations:** Department of Orthopaedic Surgery, Faculty of Medicine, Graduate School of Medicine, Hokkaido University, Kita-15 Nishi-7, Kita-ku, Sapporo, 060-8638 Japan

**Keywords:** Total hip arthroplasty, Femoral anteversion, Lower limb alignment, Knee osteoarthritis, Risk factors, Signs and symptoms, Diseases

## Abstract

Total hip arthroplasty (THA) aims to restore hip joint alignment in patients with osteoarthritis (OA) or osteonecrosis of the femoral head (ONFH). While THA directly affects hip alignment, it also influences leg and patellar alignment, potentially altering knee biomechanics and accelerating osteoarthritis progression. Postoperative alignment changes commonly include increased varus alignment in the operative leg and valgus alignment in the contralateral leg. However, the factors contributing to these changes remain unclear, particularly the role of femoral anteversion. This study analyzed 228 patients who underwent hybrid THA, assessing preoperative and postoperative radiographic parameters, including femoral anteversion, femoral offset, hip center shift, and leg length. Multiple linear regression identified factors influencing alignment changes. Femoral anteversion emerged as a key independent determinant affecting valgus alignment of the operative leg and lateral patellar tilt. Increased femoral offset and diagnosis of hip OA were also associated with alignment changes, while contralateral alignment was influenced by ipsilateral HKAA changes and patient age. These findings suggest that increased femoral anteversion following THA may mitigate excessive varus loading and protect against secondary knee OA. Accurate control of anteversion, supported by navigation or artificial intelligence (AI)-assisted planning, could optimize postoperative biomechanical balance and long-term outcomes.

## Introduction

The aim of total hip arthroplasty (THA) is to reconstruct the anatomical alignment of the hip joint compromised by osteoarthritis (OA) or osteonecrosis of the femoral head (ONFH)^1^. THA not only influences hip alignment but also affects leg and patellar alignment on both the ipsilateral and contralateral sides^2–5^. Changes in these alignments around the knees following THA may be detrimental to postoperative outcomes and may increase the risk of knee OA progression^6,7^.

Following THA, alignment changes commonly include a varus alignment in the ipsilateral leg and a valgus alignment in the contralateral leg^8–10^. Previous studies have identified several parameters that may contribute to changes in postoperative leg alignment. Factors such as medial shift of the hip center, increased femoral offset, and leg lengthening have been associated with varus changes in the ipsilateral leg, indicated by an increased hip-knee-ankle angle (HKAA)^8,10^. However, the decisive factors determining postoperative leg alignment remain unclear because of the variability in factors reported across different studies. Moreover, the parameters influencing contralateral leg-alignment changes have not been identified. This uncertainty may be due to unidentified factors, or to the multifactorial nature of parameters associated with postoperative alignments.

Leg rotation has been shown to correlate with varus/valgus leg alignment in whole-leg anteroposterior radiographs under healthy conditions^11,12^. During THA, the anteversion of a cemented stem can be easily modified based on combined anteversion theory, which in turn can alter femoral rotation^13^. Thus, changes in anteversion might be a novel contributing factor to changes in leg alignment through affecting leg rotation.

Femoral anteversion is defined as the angle between the long axis of the femoral neck and the tangent line of the posterior femoral condyles on the axial plane, and the physiological range between 5° and 20°^14^. Slipped capital femoral epiphysis^15^ and Perthes disease^16^ have been reported to be associated with increased femoral anteversion. Femoral anteversion in developmental dysplasia of the hip tends to be higher than normal, but with large variability^17^. Abnormal femoral anteversion that markedly deviates from the physiological range (5°–20°) has been associated with impaired lower-limb biomechanics including change in lever arms of muscles and ligaments^15,18^, resulting in altered contact forces in the hip joints as well as the whole lower extremity^14,19^. Especially, excessive femoral anteversion is associated with hip abductor weakness, whereas retroversion is linked to increased varus thrust resulting from increased lever arm of abductor muscle^14^. These findings suggest that the change in femoral anteversion affects postoperative leg alignment. However, no previous study has directly evaluated the femoral anteversion as a determinant factor of lower-limb alignment compared with other factors using the multivariable analysis.

Our hypothesis is that femoral anteversion is an unrecognized key contributor to lower-limb alignment. This study aimed to identify independent determinants of postoperative HKAA and lateral patellar tilt on the operative and contralateral sides. We evaluated changes in femoral anteversion, hip center, femoral offset, and leg length as candidate independent determinants using multivariable linear regression.

## Methods

### Study design and participants

**Fig. 1:**
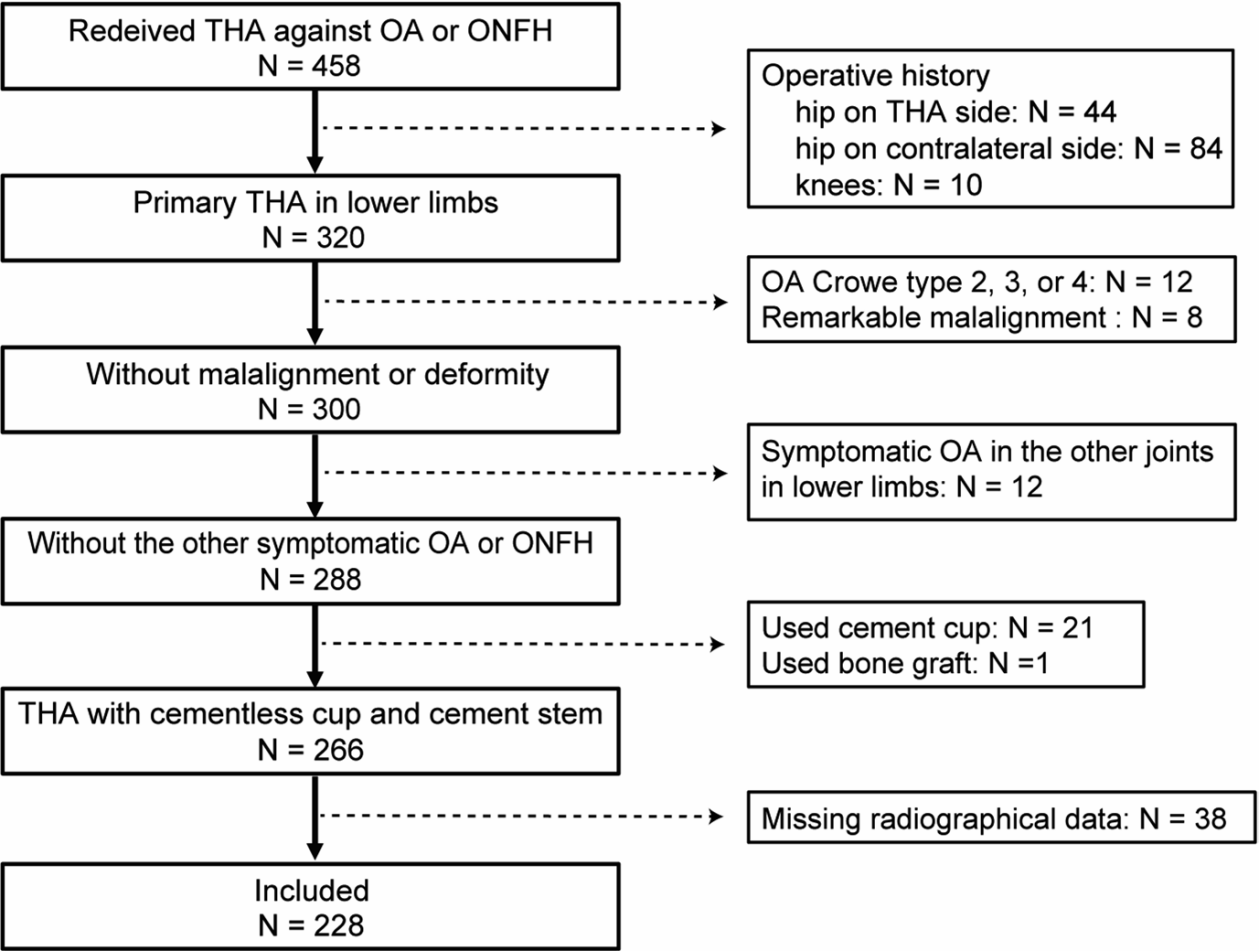
Flowchart of study enrollment. Abbreviations: OA, osteoarthritis of hip.

Our study was performed in accordance with the relevant guidelines of Hokkaido University Hospital and was approved by its Research Ethics Review Committee. Our research protocol for human samples used in this study was approved by the Research Ethics Review Committee of Hokkaido University Hospital (approval ID: 0021–0129). Informed consent for using samples in our research was obtained from all participants. Inclusion criteria comprised patients with symptomatic OA or ONFH who had undergone hybrid THA (performed by the two authors, T.S and D.T) from January 2014 to December 2022 (*n* = 458 patients, Fig. 1). Exclusion criteria comprised patients with: (i) a history of surgery on the lower limbs (*n* = 138); (ii) Crowe type 2, 3, or 4 deformities (*n* = 12); (iii) pronounced malalignment owing to deformity or severe OA in other lower limb joints (*n* = 8); (iv) symptomatic OA in other lower limb joints (*n* = 12); (v) the use of a cement cup or bone graft in THA (*n* = 22); and (vi) missing radiographic data (*n* = 38).

### Surgical indications and THA technique

THA was indicated for patients with symptomatic OA classified as Kellgren and Lawrence (KL) grade 3 or 4, and symptomatic ONFH classified as Ficat and Arlet stage 3 or 4. All procedures were performed using a posterolateral approach, with the patient positioned laterally. A porous-coated cementless cup was inserted following acetabular reaming, and a polished cemented stem was implanted using a fourth-generation cementing technique^20^.

### Demographic data

Demographic data, including age, sex, body mass index (BMI), diagnosis, classification, and components used in THA, were collected from medical records (Table 1). The mean age was 62.6 years, with 167 of 228 patients being women. Among the patients, 158 were diagnosed with OA and 70 with ONFH. All acetabular components used were porous-coated cementless cups, while all femoral components were polished-tapered cemented stems.

**Table 1:** Demographics data of the patients.

Characteristics	Value
Number of joints: patients	228: 228
Age, years	62.9 (13.1)
Female: Male	167: 61
BMI, kg/m^2^	24.8 (4.7)
Diagnosis and classification	
Osteoarthritis, joints	158
KL grade 3: grade 4	29: 129
Osteonecrosis of femoral head, joints	70
Ficat stage 3: stage 4	55: 15
Component	
Stryker Trident cup	191
Nakashima GS cup	33
Nakashima Anasta cup	4
Nakashima VLIAN stem	165
Stryker Exeter stem	63

Data show the number or mean (standard deviation). BMI, body mass index: KL, Kellgren and Lawrence classification: Exeter stem, Stryker, Kalamazoo, Michigan, USA: VLIAN stem, Teijin Nakashima Medical, Okayama, Japan: GS cup, Teijin Nakashima Medical, Okayama, Japan: Trident cup, Stryker, Kalamazoo, Michigan, USA: Anasta cup, Teijin Nakashima Medical, Okayama, Japan: Trident cup, Stryker, Kalamazoo, Michigan, USA.

### Radiological measurements

Bilateral whole-leg radiographs in the standing position and computed tomography (CT) scans from the hip to the knee were obtained two weeks preoperatively and postoperatively using an Aquilion One/ViSION Edition (Toshiba Medical Systems, Japan) with a pixel matrix of 512 × 512. The slice thickness and interval were set at 1 mm each. The images were assessed with standard angle/distance tools in picture archiving and communication system (PACS). All measurements were performed manually by two trained orthopedic surgeons (S.Y and H.I).

**Fig. 2:**
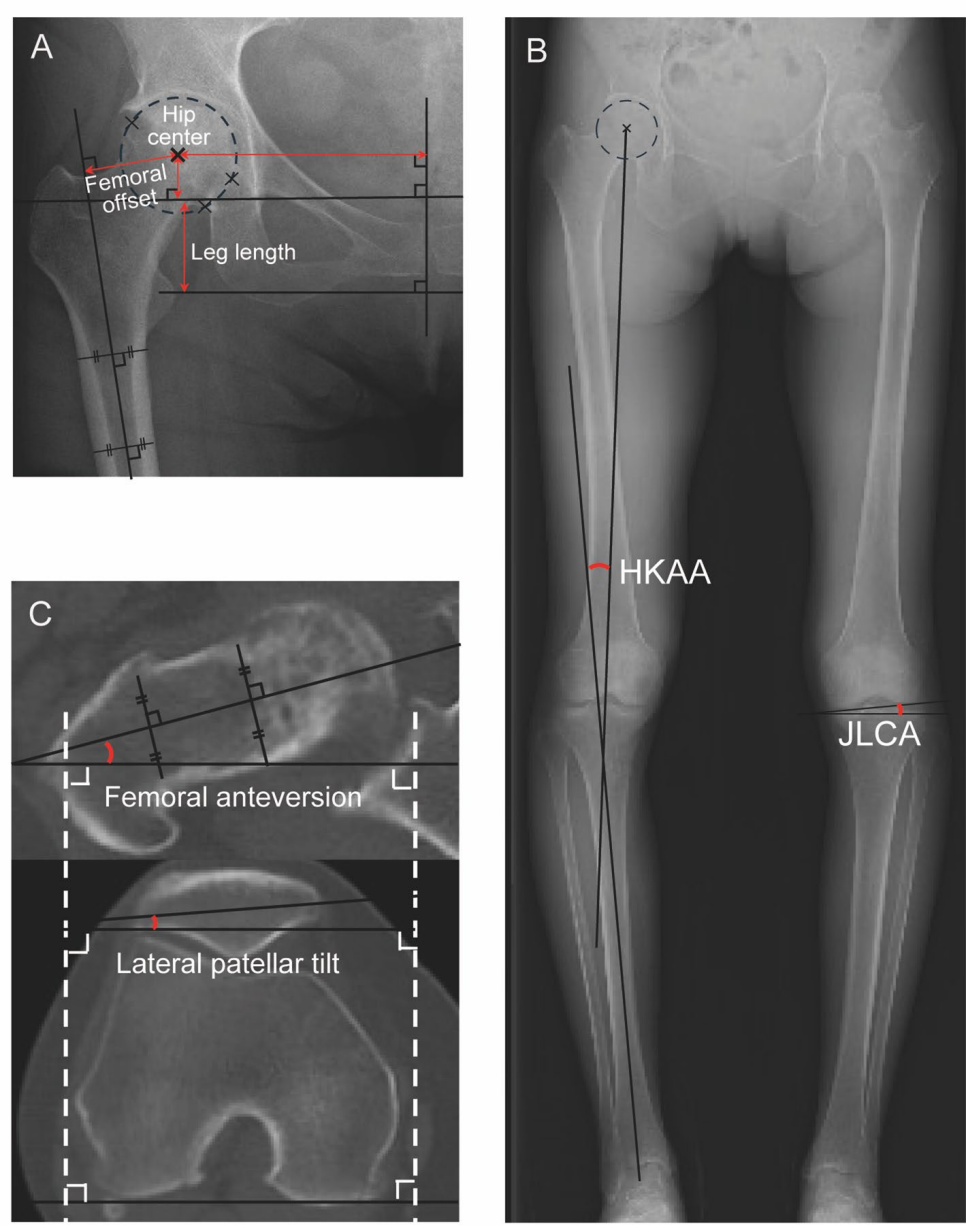
Radiological measurements. (A) Anteroposterior radiograph shows the measurement of hip center, femoral offset, and leg length. The hip center was defined as the center of circle passing through three points set arbitrarily in the intact femoral head. The height and width of hip center were measured as the perpendicular distance from the bilateral teardrops and from the bisector of the teardrops respectively. Femoral offset was measured as the perpendicular distance between the center of the femoral head and the proximal axis of the femur. Apex of the lesser trochanter was used as index of the leg length. (B) Whole-leg radiograph shows the indices used for measuring hip-knee-ankle angle (HKAA) and joint line convergence angle (JLCA). (C) Axial CT image shows the measurement of the femoral anteversion, and lateral patellar tilt based on the posterior femoral condyles.

Radiographic measurements around the hip included the height of the hip center, lateral width to the hip center, femoral offset, and leg length (Fig. 2-A). The hip center was defined as the center of a circle passing through three arbitrarily set points on the intact femoral head. The height of the hip center was measured as the vertical distance from the center of the femoral head perpendicular to the line between the bilateral teardrops. The lateral width to the hip center was measured from the center of the femoral head to the perpendicular bisector of the teardrops. Femoral offset was defined as the perpendicular distance between the center of the femoral head and the proximal femur axis. Leg length was measured as the perpendicular distance from the trans-teardrop line to the apex of the lesser trochanter.

From bilateral whole-leg radiographs in the standing position, the HKAA and joint line convergence angle (JLCA) were measured (Fig. 2-B). The HKAA was defined as the angle formed by a line drawn from the center of the femoral head through the center of the femoral condyles and a line drawn from the center of the talus through the center of the tibial spines. HKAA values were considered positive for varus alignment. The JLCA was measured as the angle between the femoral and tibial joint lines in the frontal plane, with lateral opening designated as a positive value.

Axial CT images were used to measure femoral anteversion and lateral patellar tilt (Fig. 2-C). Femoral anteversion was defined as the angle between the femoral neck axis and a line drawn along the posterior femoral condyles. Lateral patellar tilt was defined as the angle between a line connecting the medial and lateral edges of the patella and a line along the posterior femoral condyles. Interobserver reliability was assessed using the intraclass correlation coefficient (ICC), which ranged from 0.84 to 0.89, indicating excellent reproducibility.

### Data analyses

Continuous variables were described using mean and standard deviation values. A paired *t*-test was used to compare preoperative and postoperative radiographic parameters. Linear regression analyses were conducted to assess the association of various factors with changes in the HKAA and lateral patellar tilt on both operative and contralateral sides. Parameters for multiple linear analysis were selected a priori based on previous literature and clinical relevance, including age, sex, diagnosis, shift of hip center, femoral offset, leg lengthening, the JLCA, and femoral anteversion. All statistical analyses were performed using GraphPad Software version 10.2.3 (347) (GraphPad Software Inc., San Diego, CA, USA). A *P*-value < 0.05 was considered statistically significant.

### Ethics approval

This study was performed in accordance with the ethical standards laid down in the 1964 Declaration of Helsinki and its later amendments and approved by the Hokkaido University Hospital Institutional Review Board (0021–0129).

### Informed consent

Due to the retrospective nature of the study, the Hokkaido University Hospital Institutional Review Board waived the need of obtaining informed consent.

## Results

### Changes in parameters preoperative and postoperative THA

Preoperative and postoperative radiological parameters are presented in Table 2. The hip center shifted in both distal and medial directions. Increases were observed in femoral offset, leg length, femoral anteversion, and lateral patellar tilt. Lower limb alignment changed to a varus direction on the operative side, as indicated by the HKAA and the JLCA, while alignment changed to a valgus direction on the contralateral side. Changes in the JLCA and lateral patellar tilt on the contralateral side were noted but were less pronounced compared to the operative side. All these changes between preoperative and postoperative values were statistically significant.

**Table 2:** Change in parameters before and after THA.

Parameter	Preoperative	Postoperative	Value changed	*P*-value
Height of hip center, mm	26.11 ± 7.51	20.97 ± 4.44	5.14 ± 6.56	< 0.001
Lateral width of hip center, mm	101.57 ± 8.31	91.00 ± 5.89	10.56 ± 7.67	< 0.001
Femoral offset, mm	35.55 ± 8.23	41.03 ± 6.05	5.71 ± 7.62	< 0.001
Leg length discrepancy, mm	−4.53 ± 7.00	6.00 ± 6.79	10.45 ± 6.15	< 0.001
HKAA, °	0.17 ± 3.06	2.06 ± 3.43	1.89 ± 2.55	< 0.001
JLCA, °	0.05 ± 1.43	0.92 ± 1.50	0.86 ± 1.53	< 0.001
Femoral anteversion, °	21.83 ± 11.3	30.14 ± 8.21	8.31 ± 10.50	< 0.001
Lateral patellar tilt, °	11.6 ± 4.79	15.54 ± 5.70	3.93 ± 4.35	< 0.001
Contralateral HKAA, °	2.17 ± 3.02	1.91 ± 2.91	−0.25 ± 1.98	0.048
Contralateral JLCA, °	0.96 ± 1.25	0.72 ± 1.31	−0.25 ± 1.25	0.004
Contralateral lateral patellar tilt, °	11.75 ± 4.89	12.11 ± 5.04	0.40 ± 2.62	0.041

Data show the mean ± standard deviation. THA, total hip arthroplasty; HKAA, hip-knee-ankle angle: JLCA; joint line convergence angle.

### Contributing factors to changes in the HKAA on the operative THA side

Univariate linear analysis showed that diagnosis, medial shift of the hip center, changes in femoral offset, changes in the JLCA, and changes in femoral anteversion were significantly associated with changes in the HKAA on the operative side (Table 3). In multiple linear analysis, diagnosis, changes in femoral offset, and changes in femoral anteversion were identified as independent contributing factors to changes in the HKAA (Table 3).

**Table 3:** Univariate and multiple linear regression analysis for change in HKAA.

Variables	B	95% CI	b	*P*-value
Univariate linear regression				
Age	−0.002	[−0.028 to 0.023]	−0.012	0.854
Sex (Female)	0.492	[−0.261 to 1.245]	0.085	0.200
BMI	−0.069	[−0.139 to 0.001]	−0.129	0.051
Diagnosis (OA)	1.463	[0.763 to 2.162]	0.264	< 0.001
Distal shift of hip center	0.038	[−0.012 to 0.089]	0.098	0.139
Medial shift of hip center	0.057	[0.014 to 0.099]	0.167	0.010
Change in femoral offset	0.078	[0.035 to 0.120]	0.232	< 0.001
Change in global offset	0.012	[−0.022 to 0.046]	0.047	0.482
Leg lengthening	0.002	[−0.052 to 0.056]	−0.005	0.942
Change in JLCA	0.816	[0.626 to 1.007]	0.489	< 0.001
Change in femoral anteversion	−0.101	[−0.130 to −0.072]	−0.416	< 0.001
Multiple linear regression				
Age	−0.001	[−0.026 to 0.024]	−0.004	0.953
Sex (Female)	−0.504	[−1.22o to 0.212]	−0.087	0.167
Diagnosis (OA)	1.170	[0.404 to 1.936]	0.211	0.003
Distal shift of hip center	0.034	[−0.031 to 0.010]	0.088	0.305
Medial shift of hip center	0.021	[−0.024 to 0.065]	0.014	0.363
Change in femoral offset	0.043	[0.002 to 0.084]	0.127	0.041
Leg lengthening	−0.040	[−0.108 to 0.030]	−0.094	0.266
Change in femoral anteversion	−0.091	[−0.121 to −0.060]	−0.373	< 0.001

HKAA, hip-knee-ankle angle: B, unstandardized regression coefficient: CI, confidence interval: β, standardized partial regression coefficient: BMI, body mass index: OA, osteoarthritis of hip: JLCA, joint line convergence angle.

### Contributing factors to changes in the HKAA on the contralateral side

Univariate linear analysis indicated that age, changes in the contralateral JLCA, and changes in the ipsilateral HKAA were significantly associated with changes in the HKAA on the contralateral side (Table 4). In multiple linear analysis, age and changes in the ipsilateral HKAA were identified as independent contributing factors to changes in the contralateral HKAA (Table 4).

**Table 4:** Univariate and multiple linear regression analysis for change in contralateral HKAA.

Variables	B	95% CI	b	*P*-value
Univariate linear regression				
Age	0.028	[0.008 to 0.047]	0.183	0.006
Sex (Female)	−0.231	[−0.816 to 0.355]	−0.052	0.438
BMI	0.010	[−0.045 to 0.064]	0.023	0.724
Diagnosis (OA)	−0.005	[−0.567 to 0.558]	−0.001	0.987
Distal shift of hip center	−0.016	[−0.056 to 0.023]	−0.055	0.412
Medial shift of hip center	−0.018	[−0.052 to 0.016]	−0.070	0.289
Change in femoral offset	−0.027	[−0.061 to 0.007]	−0.103	0.122
Change in global offset	−0.005	[−0.031 to 0.021]	−0.026	0.712
Leg lengthening	−0.005	[−0.047 to 0.038]	−0.014	0.832
Change in contralateral JLCA	0.457	[0.257 to 0.656]	0.288	< 0.001
Change in HKAA	−0.290	[−0.455 to −0.125]	−0.374	< 0.001
Change in femoral anteversion	0.015	[−0.010 to 0.040]	0.079	0.233
Multiple linear regression				
Age	0.026	[0.005 to 0.048]	0.175	0.016
Sex (Female)	−0.149	[−0.766 to 0.468]	−0.033	0.635
Diagnosis (OA)	0.077	[−0.594 to 0.748]	0.018	0.821
Distal shift of hip center	−0.009	[−0.066 to 0.047]	−0.031	0.745
Medial shift of hip center	−0.006	[−0.044 to 0.032]	−0.022	0.766
Change in femoral offset	−0.005	[−0.041 to 0.030]	−0.021	0.762
Leg lengthening	0.007	[−0.052 to 0.067]	0.023	0.804
Change in HKAA	−0.185	[−0.299 to −0.070]	−0.238	0.002
Change in femoral anteversion	−0.011	[−0.039 to 0.017]	−0.060	0.429

HKAA, hip-knee-ankle angle: B, unstandardized regression coefficient: CI, confidence interval: β, standardized partial regression coefficient: BMI, body mass index: OA, osteoarthritis of hip: JLCA, joint line convergence angle.

### Contributing factors to changes in lateral patellar Tilt on the operative THA side

Univariate linear analysis findings indicated that distal shift of the hip center, leg lengthening, and changes in femoral anteversion were significantly associated with changes in lateral patellar tilt on the operative side (Table 5). In multiple linear analysis, changes in femoral anteversion were identified as the independent contributing factor to changes in lateral patellar tilt (Table 5).

**Table 5:** Univariate and multiple linear regression analysis for change in lateral patellar tilt.

Variables	B	95% CI	b	*P*-value
Univariate linear regression				
Age	0.016	[−0.027 to 0.059]	0.0480	0.470
Sex (Female)	−0.057	[−1.344 to 1.229]	−0.006	0.930
BMI	−0.045	[−0.165 to 0.074]	−0.050	0.456
Diagnosis (OA)	0.572	[0.661 to 1.804]	0.061	0.362
Distal shift of hip center	0.132	[0.047 to 0.217]	0.199	0.003
Medial shift of hip center	0.061	[0.013 to 0.135]	0.107	0.106
Change in femoral offset	−0.019	[−0.094 to 0.055]	−0.034	0.608
Change in global offset	−0.053	[−0.106 to 0.001]	−0.121	0.050
Leg lengthening	−0.158	[0.068 to 0.248]	0.224	< 0.001
Change in JLCA	−0.105	[−0.477 to 0.267]	−0.037	0.579
Change in HKAA	−0.214	[−0.435 to 0.008]	−0.126	0.058
Change in femoral anteversion	0.204	[0.157 to 0.251]	0.493	< 0.001
Multiple linear regression				
Age	−0.009	[−0.050 to 0.032]	−0.027	0.661
Sex (Female)	0.985	[−0.189 to 2.158]	0.100	0.100
Diagnosis (OA)	0.626	[−0.649 to 1.902]	0.066	0.334
Distal shift of hip center	0.096	[−0.002 to 0.193]	0.144	0.055
Medial shift of hip center	0.008	[−0.065 to 0.081]	0.014	0.825
Change in femoral offset	0.024	[−0.044 to 0.091]	0.042	0.488
Leg lengthening	0.031	[−0.057 to 0.119]	0.044	0.488
Change in HKAA	0.101	[−0.117 to −0.318]	0.059	0.363
Change in femoral anteversion	0.235	[0.182 to 0.289]	0.569	< 0.001

B, unstandardized regression coefficient; CI, confidence interval: β, standardized partial regression coefficient: BMI, body mass index; OA, osteoarthritis of hip: HKAA, hip-knee-ankle angle: JLCA, joint line convergence angle.

### Contributing factors to changes in lateral patellar tilt on the contralateral side

Changes in lateral patellar tilt on the ipsilateral side were identified as contributing factors to changes in lateral patellar tilt on the contralateral side in both univariate and multiple linear analysis (Table 6).

**Table 6:** Univariate linear regression analysis for change in contralateral patellar tilt.

Variables	B	95% CI	b	*P*-value
Univariate linear regression				
Age	0.001	[−0.025 to 0.027]	0.005	0.934
Sex (Female)	−0.175	[−0.952 to 0.602]	−0.030	0.657
BMI	−0.057	[−0.129 to 0.015]	−0.103	0.121
Diagnosis (OA)	0.130	[−0.616 to 0.876]	0.023	0.732
Distal shift of hip center	−0.024	[−0.076 to 0.028]	−0.060	0.368
Medial shift of hip center	−0.015	[−0.060 to 0.030]	−0.044	0.509
Change in femoral offset	−0.008	[−0.053 to 0.037]	−0.024	0.128
Change in global offset	0.007	[−0.025 to 0.039]	0.026	0.674
Leg lengthening	−0.025	[−0.081 to 0.031]	−0.058	0.386
Change in JLCA	0.093	[−0.132 to 0.318]	0.054	0.415
Change in HKAA	0.081	[−0.053 to 0.216]	0.079	0.234
Change in contralateral JLCA	0.069	[−0.207 to 0.345]	0.033	0.622
Change in contralateral HKAA	0.165	[−0.008 to 0.337]	0.124	0.061
Change in femoral anteversion	0.031	[−0.002 to 0.063]	0.122	0.065
Change in lateral patellar tilt	0.120	[0.042 to 0.198]	0.199	0.003
Multiple linear regression				
Age	−0.012	[−0.041 to 0.018]	−0.058	0.433
Sex (Female)	−0.100	[−0.931 to 0.731]	−0.017	0.813
Diagnosis (OA)	0.392	[−0.496 to 1.279]	0.069	0.385
Distal shift of hip center	−0.014	[−0.083 to 0.055]	−0.035	0.689
Medial shift of hip center	−0.015	[−0.067 to 0.036]	−0.045	0.561
Change in femoral offset	0.002	[−0.457 to 0.049]	0.005	0.942
Leg lengthening	−0.032	[−0.046 to 0.049]	−0.074	0.942
Change in contralateral HKAA	0.116	[−0.062 to 0.295]	0.088	0.201
Change in femoral anteversion	0.009	[−0.032 to 0.050]	0.035	0.675
Change in lateral patellar tilt	0.119	[0.023 to 0.215]	0.197	0.015

B, unstandardized regression coefficient: CI, confidence interval: β, standardized partial regression coefficient: BMI, body mass index: OA, osteoarthritis of hip: HKAA, hip-knee-ankle angle: JLCA, joint line convergence angle.

## Discussion

In this retrospective study, we comprehensively analyzed alignment changes after hybrid THA and investigated independent contributing factors for both the operative and contralateral lower limbs using a multivariate approach. Through multivariate regression, three parameters—femoral anteversion, diagnosis of hip OA, and femoral offset—were identified as independent determinants of operative-side HKAA. Importantly, this study evaluated contralateral alignment as well and revealed that operative-side changes influenced the opposite limb alignment, suggesting the bilateral interdependence of alignment after THA.

In the present study, characteristic postoperative patterns—such as medial and distal translation of the hip center, increased femoral offset, mild leg lengthening, increased femoral anteversion, varus shift of operative leg alignment, and increased lateral patellar tilt—were consistently observed, in line with previous reports describing typical morphological adjustments following THA^8,10,11^. These findings confirm that the present cohort reflects the general biomechanical consequences of THA, providing a valid foundation for subsequent analyses of alignment determinants.

### Operative-side alignment changes

Femoral anteversion increase was associated with a valgus shift, whereas osteoarthritis diagnosis and increased femoral offset independently contributed to a varus shift. Although hip-center medialization has been reported to contribute to varus alignment^8^, it was not identified as an independent determinant in our multivariable analysis.

#### Femoral anteversion

Among the analyzed parameters, femoral anteversion had the greatest influence on postoperative alignment (largest absolute β). Increased femoral anteversion is suggested to influence lower limb alignment through internal rotation of the leg, which biomechanically induces a valgus shift (decrease in HKAA)^11,12^. This finding suggests that increased anteversion may function as a counteracting factor that prevents excessive varus deformity after THA. Excessive varus alignment is well known to accelerate the progression of knee OA^21,22,23^, whereas valgus realignment procedures such as high tibial osteotomy (HTO) and distal femoral osteotomy (DFO) effectively slow its development^24,25^. Similarly, previous biomechanical studies have reported reduced tibiofemoral contact forces in the medial compartment after THA^26^, which can partly explain the lower incidence of knee OA following the procedure^27^. Taken together, these findings indicate that increased femoral anteversion, as the primary valgus-inducing factor, contributes to maintaining optimal postoperative alignment by mitigating varus loading on the lower limb.

#### Hip center medialization

Previous studies have reported that medialization of the hip center is associated with varus alignment^8^. However, the study was limited by smaller sample sizes and relied primarily on univariate analyses. In the present study, although hip-center medialization showed a correlation in univariate analysis, it was not identified as an independent determinant in the multivariate model. Although medialization increases the hip–knee distance, a shortened abductor lever arm may reduce the lateral force on the proximal femur^28^ and counteract its effect toward varus alignment.

#### Femoral offset

The observed association between increased femoral offset and varus alignment is consistent with previous reports^8,10^. This relationship suggests that an increased abductor lever arm and a widened hip–knee distance promote a varus shift in lower limb alignment^28^.

#### Leg length change

Reports on the relationship between leg length change and limb alignment after THA are limited and mostly based on univariate analyses^8^. Although the operative leg generally lengthens after THA, the direction of alignment change—toward varus or valgus—appears to depend more on biomechanical factors such as stress distribution and the balance of muscle and ligament tension than on leg length itself.

#### Diagnosis of hip OA

Few studies have mentioned diagnosis hip OA is a determinant of postoperative lower-limb alignment following THA^11^. In the present study, hip OA was identified as an independent factor associated with a varus shift of the operative limb alignment. Patients with hip OA tend to suffer from prolonged joint deterioration, pain, and restricted range of motion, resulting in muscle weakness on the affected leg^22–25^. This muscle weakness and imbalance may increase susceptibility to postoperative changes in leg alignment.

### Contralateral alignment changes

Aging and a varus shift on the operative side were independently associated with a valgus change in the contralateral limb. Furthermore, operative-side lateral patellar tilt was the only determinant of contralateral lateral patellar tilt.

#### Age

Aging is known to cause systemic muscle weakness, ligament laxity, and balance deficits^29,30^. These age-related musculoskeletal changes may make the contralateral weight-bearing alignment more susceptible to the influence of the operative side.

### Operative HKAA

#### Operative lateral patellar tilt

Operative-side parameters (HKAA and lateral patellar tilt) influenced the corresponding parameters on the contralateral side. This finding suggests that changes in limb alignment and patellar tracking occur in an interdependent manner, likely mediated by neuromechanical coupling and compensatory neuromuscular mechanisms between both limbs^31^.

### Clinical implications

Femoral anteversion emerged as a key determinant of postoperative lower limb alignment, and increased anteversion appeared to help prevent excessive varus loading, which is associated with knee OA progression. Intraoperatively, femoral anteversion can be adjusted intraoperatively by rotating the cemented stem relative to the tibial axis, using a measuring device attachable to rasp handles^32,33^ or robotic navigation^34^. The determination of anteversion must consider factors such as range of motion, impingement, and dislocation resistance^35–38^. Although abnormal femoral anteversion beyond the physiological range of 5°–20° has been associated with impaired lower-limb biomechanics^14,15,18^, a slight increase up to approximately 25°–30° can be tolerated without compromising hip stability, provided that the combined anteversion remains within the functional safe zone^39–41^. Taken together, current evidence suggests that, within biomechanically safe limits, increasing femoral anteversion may mitigate excessive varus loading and thereby help optimize postoperative lower-limb alignment. Based on the present findings, artificial intelligence (AI)-assisted navigation may help determine precise implant positioning to achieve patient-specific alignment targets and optimize surgical outcomes in the future. Conversely, in patients with preexisting excessive femoral anteversion beyond the physiological range, further increasing anteversion intraoperatively is technically difficult because of impingement-related constraints. Therefore, these patients may have a higher risk of postoperative varus shift in the operative limb alignment and progression of ipsilateral knee OA. Although alignment was assessed at only two weeks postoperatively, this early evaluation provides insight into the pure biomechanical effects of implant positioning on both the operative and contralateral limbs, independent of functional muscle compensation. Predicting the postoperative mechanical baseline may help develop individualized preoperative rehabilitation strategies and facilitate continuous postoperative monitoring of both operative and contralateral limbs to improve long-term outcomes.

### Limitation

While this study provides valuable insights, several limitations should be acknowledged. The first limitation of this study is that postoperative measurements were performed only two weeks after surgery. Long-term follow-up is needed to determine whether postoperative alignment continues to shift toward excessive varus or valgus, and whether such changes contribute to poor outcomes, including progressive knee degeneration^42–44^. In addition, the retrospective design may have introduced selection bias, and the exclusion of patients with severe deformities or prior lower limb surgeries limits the generalizability of the findings. Future prospective studies with larger cohorts, including patients with varying degrees of deformity and different surgical approaches, are needed to validate our findings.

### Conclusion

In conclusion, femoral anteversion was identified as a major determinant of postoperative alignment and may serve as a protective factor against excessive varus loading. Additionally, the study findings suggest that changes in the operative leg can affect the contralateral leg, emphasizing the interconnected nature of lower limb biomechanics. Within the physiological and biomechanically safe range, increased femoral anteversion may help optimize lower-limb alignment and potentially reduce the risk of subsequent knee osteoarthritis.

## Data Availability

The datasets generated during and/or analysed during the current study are available from the corresponding author on reasonable request.
